# Novel Anti-Alzheimer's Therapeutic Molecules Targeting Amyloid Precursor Protein Processing

**DOI:** 10.1155/2020/7039138

**Published:** 2020-04-29

**Authors:** Md. Sahab Uddin, Md. Tanvir Kabir, Philippe Jeandet, Bijo Mathew, Ghulam Md Ashraf, Asma Perveen, May N. Bin-Jumah, Shaker A. Mousa, Mohamed M. Abdel-Daim

**Affiliations:** ^1^Department of Pharmacy, Southeast University, Dhaka, Bangladesh; ^2^Pharmakon Neuroscience Research Network, Dhaka, Bangladesh; ^3^Department of Pharmacy, Brac University, Dhaka, Bangladesh; ^4^Research Unit, Induced Resistance and Plant Bioprotection, EA 4707, SFR Condorcet FR CNRS 3417, Faculty of Sciences, University of Reims Champagne-Ardenne, PO Box 1039, 51687 Reims Cedex 2, France; ^5^Division of Drug Design and Medicinal Chemistry Research Lab, Department of Pharmaceutical Chemistry, Ahalia School of Pharmacy, Palakkad, India; ^6^King Fahd Medical Research Center, King Abdulaziz University, Jeddah 21589, Saudi Arabia; ^7^Department of Medical Laboratory Technology, Faculty of Applied Medical Sciences, King Abdulaziz University, Jeddah 21589, Saudi Arabia; ^8^Glocal School of Life Sciences, Glocal University, Saharanpur, India; ^9^Department of Biology, College of Science, Princess Nourah bint Abdulrahman University, Riyadh 11474, Saudi Arabia; ^10^Pharmaceutical Research Institute, Albany College of Pharmacy and Health Sciences, New York, NY 12144, USA; ^11^Department of Zoology, College of Science, King Saud University, P.O. Box 2455, Riyadh 11451, Saudi Arabia; ^12^Pharmacology Department, Faculty of Veterinary Medicine, Suez Canal University, Ismailia 41522, Egypt

## Abstract

Alzheimer's disease (AD) is the most common cause of dementia among older people, and the prevalence of this disease is estimated to rise quickly in the upcoming years. Unfortunately, almost all of the drug candidates tested for AD until now have failed to exhibit any efficacy. Henceforth, there is an increased necessity to avert and/or slow down the advancement of AD. It is known that one of the major pathological characteristics of AD is the presence of senile plaques (SPs) in the brain. These SPs are composed of aggregated amyloid beta (A*β*), derived from the amyloid precursor protein (APP). Pharmaceutical companies have conducted a number of studies in order to identify safe and effective anti-A*β* drugs to combat AD. It is known that *α*-, *β*-, and *γ*-secretases are the three proteases that are involved in APP processing. Furthermore, there is a growing interest in these proteases, as they have a contribution to the modulation and production of A*β*. It has been observed that small compounds can be used to target these important proteases. Indeed, these compounds must satisfy the common strict requirements of a drug candidate targeted for brain penetration and selectivity toward different proteases. In this article, we have focused on the auspicious molecules which are under development for targeting APP-processing enzymes. We have also presented several anti-AD molecules targeting A*β* accumulation and phosphorylation signaling in APP processing. This review highlights the structure-activity relationship and other physicochemical features of several pharmacological candidates in order to successfully develop new anti-AD drugs.

## 1. Introduction

Alzheimer's disease (AD) is one of the utmost prevalent age-related neurodegenerative disorders affecting older people [1, 2]. The main risk for the development and progression of AD is age, and in people over the age of 65, the risk becomes double nearly every 5 years [3–5]. In 2015, it was estimated that around 44 million people were affected by this neurodegenerative disorder. However, this number is projected to become double by the year 2050 [6, 7]. It has been found that most of the AD patients (i.e., over 95%) have sporadic AD (SAD) or late-onset AD (LOAD), a multifactorial disease in which genetic predisposition and environmental factors have significant contribution in the pathology [8, 9]. On the other hand, less than 5% of the AD people is found to have either early-onset AD (EOAD) or familial AD (FAD). It has been found that the aforesaid forms of AD (i.e., EOAD and FAD) might take place because of the mutations in any of the presenilin-1 (*PSEN1*), presenilin-2 (*PSEN2*), and amyloid precursor protein (*APP*) genes [10–12].

The neuropathological characteristics of AD include an aberrant buildup of the amyloid beta (A*β*) peptide in amyloid plaques and aggregation of hyperphosphorylated tau in intracellular neurofibrillary tangles (NFTs) [13–16]. Furthermore, dystrophic neurites (DNs), neuropil threads, related astrogliosis, and microglial activation are found to often coexist [17, 18]. The downstream effects of these pathological mechanisms involve neurodegeneration with neuronal and synaptic loss, which can lead to macroscopic atrophy [19]. It has been suggested in various studies that AD-related alterations in the brain might start even 20 or more years before the symptoms even appear [20–23]. It has been found that when the initial alterations take place, the brain compensates for them and permits people to continue to function normally. However, when the neuronal damage continues to rise, the brain can no longer compensate for the alterations, and patients exhibit subtle cognitive impairment [24]. At a later stage, neuronal damage becomes so substantial that people exhibit clear cognitive impairment, including symptoms for instance loss of memory or confusion regarding the place or time [25, 26].

Currently, the available drugs only provide symptomatic treatment and do not have the proper ability to affect the advancement of the AD [27, 28]. Henceforth, it is essential to develop treatments that can slow, delay, or prevent the advancement of the disease and also target the AD-related pathological processes. Interestingly, it has been observed that if the onset of AD could be delayed by five years, the overall frequency of AD would be reduced by 40% [29].

Over the course of time, A*β* has been regarded as the key therapeutic target for AD [30–32]. Furthermore, a number of pharmaceutical/biopharmaceutical companies are trying to develop therapeutic compounds (i.e., as small molecules or immunotherapies) to reduce the accumulation of A*β* with possible positive action on AD pathology. In this review, we have focused on the role of APP-cleaving secretase-based novel molecules as therapeutic targets for AD. Furthermore, auspicious molecules targeting A*β* accumulation and phosphorylation signaling in APP processing are also presented.

## 2. Approved Anti-Alzheimer's Drugs

**Figure 1:**
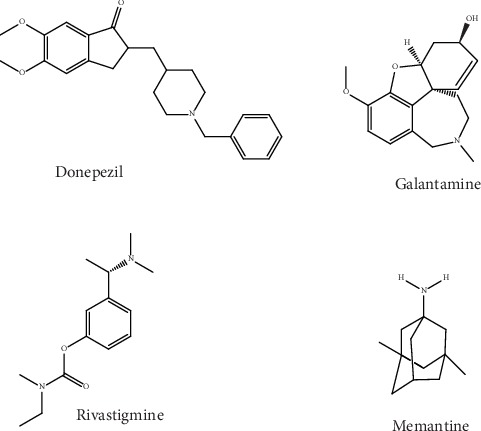
Chemical structure of approved anti-Alzheimer's drugs.

Out of the five Food and Drug Administration (FDA) -approved drugs (Figure 1) for AD therapy, four of them are acetylcholinesterase inhibitors (AChEIs) and one is N-methyl-D-aspartic acid (NMDA) receptor antagonist [33, 34]. The AChEIs (i.e., galantamine, rivastigmine, donepezil, and tacrine) have been found to play a role in improving the cholinergic neurotransmission [34, 35]. Memantine is the only noncholinergic and NMDA receptor antagonist drug. Memantine plays roles in the restoration of the A*β*-stimulated calcium imbalance (i.e., intracellular buildup of Ca^2+^) and the associated reduction of neuronal death [36]. Unfortunately, memantine and currently approved cholinesterase inhibitors (ChEIs) only provide relief of the dementia symptoms, and these agents possess limited clinical effects [37, 38]. Over the last 20 years, a number of drug candidates have been developed with the aim of hitting different and novel targets of this disease. However, effective disease-modifying therapeutic agents are yet to be found [39–41]. Other than the cholinergic recovery, researchers are also searching for other AD targets, for instance, pathogenic A*β* and tau aggregates [42, 43] as well as dysregulated metal ions [44].

## 3. Amyloid Precursor Protein Processing

**Figure 2:**
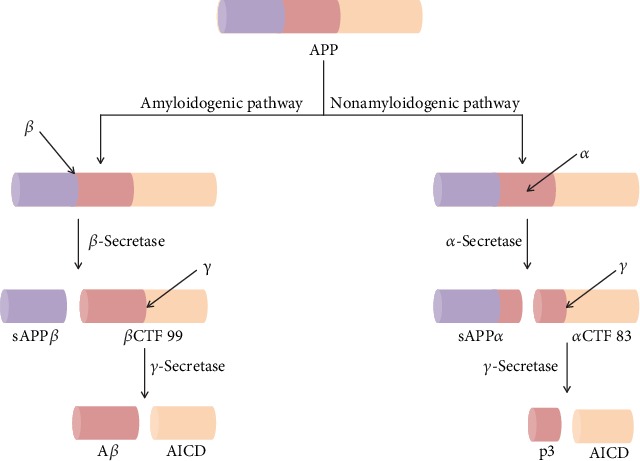
The amyloidogenic and nonamyloidogenic pathways of amyloid precursor protein processing. In the amyloidogenic pathway, cleavages of APP by *β*- and *γ*-secretases lead to the genesis of A*β* peptides. On the other hand, in the nonamyloidogenic pathway, cleavages of APP by *α*- and *γ*-secretases lead to the genesis of p3 and AICD. APP: amyloid precursor protein; sAPP*β*: soluble APP beta; *β*CTF 99: beta C-terminal fragment 99; sAPP*α*: soluble APP alpha; *α*CTF 83: alpha C-terminal fragment 83; AICD: APP intracellular domain.

APP has been found to be generated in large quantities in neurons and is metabolized very rapidly [45, 46]. In fact, APP processing is reliant on secretase enzymes (i.e. *α*-, *β*-, and *γ*-secretases), which yield products that are secreted into the extracellular space or which stay within or related to the cell. Amyloidogenic and nonamyloidogenic are the 2 pathways of APP processing [23] as shown in Figure 2. Three enzymes, which are members of the ADAM family (i.e., a disintegrin and metalloprotease), play a role in harboring the activity of *α*-secretase; these include ADAM17, ADAM10, and ADAM9 [47]. These enzymes are all primarily membrane-bound and cell-surface glycoproteins. Furthermore, they have a contribution in cell fusion, degradation of matrix protein, ectodomain shedding, and cell adhesion [48–52]. *β*-Secretase (BACE-1) is regarded as the rate-limiting enzyme in the proteolytic processing of APP. *β*-Secretase is a type I transmembrane protein and belongs to the pepsin family of aspartyl proteases [53–58]. *β*-Secretase possesses an *N*-terminal catalytic domain, harboring a transmembrane domain, two catalytic aspartic residues, and a short C-terminal cytoplasmic tail. *γ*-Secretase (GS) is a heterogeneous protein complex that possesses four transmembrane proteins including anterior pharynx-defective 1 (APH1), presenilin enhancer 2 (PEN2), nicastrin (NCT), and presenilins (PS1 and PS2) [59–61].

It has been observed that the nonamyloidogenic APP-processing pathway initiates with *α*-secretase-arbitrated APP cleavage at amino acid 687 (i.e., in the APP 770 isoform) which has been found to release the soluble APP alpha (sAPP*α*) into the extracellular space. Therefore, in the plasma membrane, a C-terminal fragment (CTF) of APP that is 83 amino acids in length (CTF 83) stays embedded. It has been found that the GS-mediated cleavage of CTF 83 then results in the APP intracellular domain (AICD) release into the cytoplasm and a small p3 fragment into the extracellular space. On the other hand, in the amyloidogenic APP processing, *β*-secretase cleaves APP which leads to the generation of the CTF that is 99 amino acids in length (CTF 99). It has also been observed that GS-mediated CTF 99 cleavage causes AICD release into the cytoplasm and A*β* into the extracellular space [62]. The activity of *α*-secretase is arbitrated via one or more enzymes from a disintegrin and metalloprotease (ADAM) family, with ADAM19, 17, 10, and 9 being the most possible candidates [48, 63, 64]. In the brain, BACE-1 is the main *β*-secretase [65], whereas GS is a multiprotein complex [66, 67].

## 4. Anti-Alzheimer's Molecules Targeting *α*-Secretase

It has been suggested by recent studies that the detected *α*-secretases exhibit an increased level of redundancy. In addition, it is still unclear exactly which *α*-secretases are accountable for cleavage of APP in neurons and other brain cells [51, 68]. Henceforth, it is essential to overcome the aforesaid ambiguity in order to develop effective compounds which will directly activate the *α*-secretases. An indirect and alternative approach to promote the *α*-secretase-arbitrated APP cleavage might be the induction of one or more of the signal transduction pathways associated with the regulation of the *α*-secretase activity. A number of pathways including mitogen-activated protein kinases, tyrosine kinases, protein kinase C, and calcium-mediated pathways have been found to play roles in terms of regulation of *α*-secretase activity. Indeed, it is possible to develop compounds that will have the capacity to induce the activity of *α*-secretase via these pathways [69]. It has been proposed that derivatives of retinoic acid have the ability to elevate the level of ADAM10 transcription. In addition to this, these derivatives might also have the potential to indirectly induce the APP cleavage mediated by *α*-secretase [70].

However, as an AD drug treatment, the development of a direct *α*-secretase activator appears unlikely, at least in the short-term. In clinical trials, the drug treatments that indirectly stimulate the activity of *α*-secretase are already being studied. Evidence reporting that the indirect activation of *α*-secretase is effectively attained through the agonists of *α*-7-nicotinic acetylcholine receptor (*α*7-nAChR), an agonist of 5-hydroxytryptamine receptor 4 (5-HT_4_) and a modulator of the gamma-aminobutyric acid (GABA) receptor, has been utilized as support to justify the clinical development of these agents [71]. However, there is a scarcity of data to support the fact that these compounds can elevate the APP cleavage mediated via *α*-secretase and decrease the levels of A*β*. But, in spite of such concerns, the approach is appealing from a theoretical perspective, according to the fact that these drugs might be approved based on their symptomatic benefits. More extensive studies can be further carried out regarding their potential to modify the disease [72–74].

### 4.1. GABA_A_ Receptor Modulators and PDE4 Inhibitors

**Figure 3:**
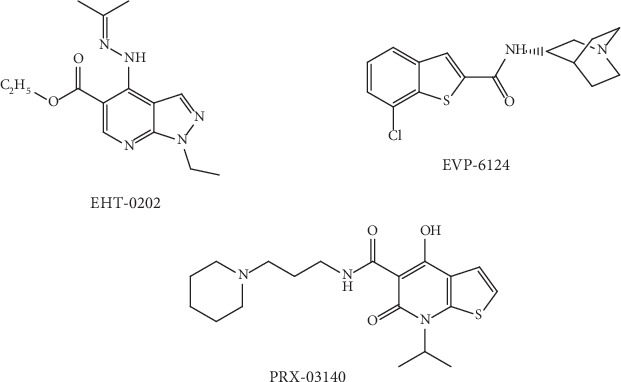
Chemical structure of auspicious molecules targeting *α*-secretase activity.

EHT-0202 (Figure 3) is a modulator of the GABA type A (GABA_A_) receptor and an inhibitor of phosphodiesterase 4 (PDE4) that has been developed as an AD treatment. EHT-0202 has the unique property of stimulating the activity of *α*-secretase via elevating the generation of procognitive and neurotrophic sAPP*α* fragments of APP. Preclinical studies revealed that EHT-0202 has the ability to protect the cortical neurons against A*β*42 and related stresses and that the provided neuroprotection is linked with the induction of sAPP*α*. In addition to this, it has also been revealed that EHT-0202 exhibits precognitive activities in several preclinical models such as memory impairment related to age and amnesia induced by scopolamine. Preclinical studies in guinea pigs and rats have shown that chronic oral administrations of EHT-0202 can lead to decreased levels of A*β*42 in cerebrospinal fluid (CSF) and brain [75]. It has been reported by Désiré et al. [76] on the identification in AD patients of blood-based transcriptomic signatures related to treatment response of EHT-0202 in a placebo-controlled, three-month, phase IIA study for estimating the exploratory efficacy, tolerability, and clinical safety of EHT-0202 (i.e., 80 and 40 mg twice a day) as adjunctive therapy to one cholinesterase inhibitor in mild-to-moderate AD individuals. Additionally, this pilot study also confirmed the value of using blood transcriptomic signatures as biomarkers for monitoring efficacy or predicting the response of patients, for a given therapeutic drug in the case of AD. For EHT-0202 or other AD drugs, such biomarkers might provide help to enhance the approaches in order to comprehend the drug mechanism efficacy, detect suitable patient populations for treatment, and/or elucidate the inherent subjectivity in most clinical endpoints utilized in Alzheimer's treatment [76].

### 4.2. Partial *α*7-nAChR Agonists

EVP-6124 (encenicline, Figure 3) is a partial agonist of *α*7-nAChR which is under study to treat AD. EVP-6124 can cause activation of *α*7-nAChR at low nanomolar brain concentrations and can also improve memory performance in rats [77]. In the case of phase I and II clinical studies performed in individuals with mild-to-moderate AD, it has been observed that the EVP-6124 treatment was tolerated and exhibited momentous enhancements as compared to placebo on functional and cognitive measures [77]. However, a clinical hold was imposed on EVP-6124 by FDA in 2015 due to the reported gastrointestinal side effects observed in two phase III Alzheimer's studies [78].

### 4.3. Partial 5-HT_4_ Receptor Agonists

Activation of 5-HT_4_ receptors can lead to possible memory improvement and also changes in brain 5-HT_4_ receptors in AD [79]. On the other hand, 5-HT_4_ receptor stimulation is found to be linked with both effects on acetylcholine release and APP processing [79]. For AD, PRX-03140 (Figure 3) is a partial agonist (i.e. 18% compared with 5-HT) of the 5-HT_4_ receptor which is being developed by EPIX Pharmaceuticals [80]. It has been observed in a phase 2a clinical trial of PRX-03140 (as a single agent and in combination with donepezil) in mild AD individuals administered daily oral 150 mg doses of PRX-03140 as monotherapy achieved a mean 3.6-point enhancement on the Alzheimer's Disease Assessment Scale-Cognitive Subscale (ADAS-Cog) versus a 0.9-point worsening in individuals receiving placebo (*P* = 0.021). When PRX-03140 was used as a monotherapy, it was found to be well tolerated. In addition to this, no significant drug-related adverse effects were observed, when PRX-03140 was used in combination with donepezil [81]. Unfortunately, PRX-00023 development was discontinued by EPIX Pharmaceuticals in March 2008 because of the lack of efficacy exhibited in a recently completed phase 2b trial in individuals with major depressive disorder [82].

### 4.4. Natural *α*-Secretase Modulators

#### 4.4.1. Retinoids

**Figure 4:**
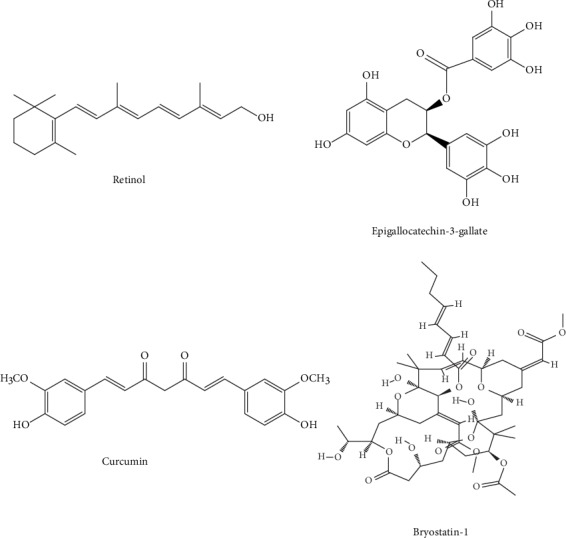
Chemical structure of auspicious natural *α*-secretase modulators targeting *α*-secretase activity.

The retinoid family involves retinol (i.e., vitamin A; Figure 4) and its natural derivatives. Retinoids have a significant contribution in regulating various activities of the adult brain including long-term potentiation, release of neurotransmitters, neurite growth, and neuronal differentiation [83]. The study of Corcoran et al. [84] first confirmed that decrease in the brain retinoid acid signaling pathway can lead to deposition of A*β* in the adult rat brain. In a different study, it was observed that tamibarotene (i.e. an agonist of the retinoid receptor) reduced the levels of A*β*_42_ and A*β*_40_ by upregulating the expression of *α*-secretase in A*β*PP23 transgenic mice [85]. Recently, Cummings et al. [86] carried out a placebo-controlled, randomized, double-blind, parallel-group study of a single dose (300 mg per day) of bexarotene (a highly specific agonist of retinoid X receptor) in individuals with AD and both noncarriers and carriers of the apolipoprotein E (*APOE*) allele. Individuals who received treatment for four weeks and were of the noncarrier group experienced decreased A*β* levels in the brain as compared to those of the *APOE4* carrier group [86]. Ghosal et al. [87], in another clinical study, assessed the activity of bexarotene in the alteration of A*β* metabolism in cognitively healthy participants. This study reported a significant problem with bexarotene treatment because of its poor CNS-penetrating capacity in cognitively healthy individuals [87].

#### 4.4.2. Epigallocatechin-3-Gallate

Epigallocatechin-3-gallate (EGCG, Figure 4) is a polyphenol found in the leaves of tea plants (i.e. *Camellia sinensis*). This polyphenol possesses antioxidant activities that may be beneficial in the case of AD as oxidative stress plays a vital role in the development of this devastating disorder [88]. A study by de la Torre et al. [89] described that when EGCG is administered in parallel with cognitive training to patients with Down's syndrome at a dose of 9 mg/kg/day for 12 months, beneficial actions were observed on memory as compared to placebo-receiving participants or the placebo plus cognitive training group in cognitive tests. Although this clinical study has been carried out in individuals displaying Down's syndrome only, in order to assess the ability of EGCG in improving cognitive functions and to observe its activities on APP and DYRK1A, the activity of this polyphenol needs also to be evaluated in the case of AD [89]. The activity of a formulation of EGCG named Sunphenon EGCg was studied in individuals with early-stage of AD in a clinical study, but till now, the findings regarding the potential disease-modifying activities have not been posted [90].

#### 4.4.3. Curcumin

Curcumin (Figure 4), the major curcuminoid of turmeric, comes from the turmeric rhizome *Curcuma longa*. Because of its small size, curcumin can easily cross the blood-brain barrier (BBB), and this natural product has been recommended as a promising therapy for AD [91]. Amino acid conjugates of curcumin were developed by Narasingapa et al. [92]. In their study, they observed that these conjugates had a potent *α*-secretase stimulatory effect [92]. The efficacy of this compound in the case of AD was also evaluated through multiple clinical studies. The potential effects of curcumin and *Ginkgo biloba* leaf extracts on AD progression have also been evaluated in a clinical study [93]. However, the findings revealed that the combination of curcumin and extracts was unsuccessful in reducing the levels of A*β* in the blood of AD-affected individuals or in improving their cognitive functions [93]. In a different clinical study, curcumin's tolerability, safety, and activity were evaluated on individuals with mild-to-moderate AD by Ringman et al. [94]. This study did not report any beneficial action of curcumin in reducing the levels of A*β* in CSF. Moreover, it was suggested that the inefficacy of curcumin may be linked to its poor bioavailability [94].

#### 4.4.4. Bryostatin-1

Bryostatin-1 (Figure 4) is a naturally occurring macrocyclic lactone derived from the marine bryozoan (i.e. *Bugula neritina*). Bryostatin-1 has the ability to induce the activity of *α*-secretase by protein kinase C (PKC) activation and subsequent elevation of sAPP*α* release [95]. Multiple clinical studies have assessed the efficacy of this compound in individuals with AD. Participants were administered a single intravenous placebo dose or 25 *μ*g/m^2^ bryostatin-1 in a single-dose randomized double-blind phase IIA clinical study [95]. In that study, pharmacodynamics, pharmacokinetics, and efficacy of bryostatin-1 were also studied. It was observed that bryostatin-1 was well tolerated in individuals with AD. This compound also increased the Mini-Mental State Examination (MMSE) score, and no adverse events were observed with the use of this compound [95]. In a different phase II clinical study, bryostatin-1 was administered intravenously at the doses of 20 and 40 *μ*g in order to treat patients with moderately severe to severe AD [96]. The primary endpoint of this study was not significant in the full data set [96].

## 5. Anti-Alzheimer's Molecules Targeting *β*-Secretase

Following the discovery of BACE-1, many attempts have been made in order to develop small-molecule BACE-1 inhibitors. Interestingly, peptide-based mimetics of the APP *β*-cleavage site were the first generation of BACE-1 inhibitors, which contained the APP *β*-site scissile amide bond replaced with a nonhydrolyzable transition state analog, for example, statin [55]. In recent times, nonpeptidergic compounds containing high affinities for BACE-1 have been produced [97, 98].

BI-1181181 is an orally active and first-generation BACE-1 inhibitor. Unfortunately, because of its low BBB penetration and low oral bioavailability, BI-1181181 has been found to fail in phase I clinical trials. Subsequently, in clinical trials, second-generation BACE-1 inhibitors including LY-2886721, LY-2811376, and RG-7129 have also failed due to liver toxicity [99, 100]. Fortunately, the third-generation BACE-1 inhibitors including JNJ-54861911, CNP-520, AZD-3293, and E-2609 have exhibited encouraging clinical data and satisfactory pharmacokinetic (PK) parameters in ongoing studies. Even though verubecestat (MK-8931) has the ability to decrease the A*β* level of CNS in AD individuals and animal models [101], nevertheless, in February 2017, Merck (an American multinational pharmaceutical company) declared that they will stop the clinical trial in mild-to-moderate AD individuals due to its lack of efficacy [102]. However, APECS trials in individuals with prodromal AD are still ongoing.

As a disease-modifying AD treatment, CTS-21166 is a small-molecule BACE-1 inhibitor that is being developed. It has been reported that CoMentis (an American biotech company) has started a phase I clinical trial of its orally bioavailable small-molecule CTS-21166. As mentioned by CoMentis, CTS-21166 is a highly selective, efficacious, and potent brain-penetrating BACE-1 inhibitor [103]. Nonetheless, initial clinical outcomes of CTS-21166 were found to be unsatisfactory [104, 105].

### 5.1. Acyl Guanidine-Based Inhibitors

**Figure 5:**
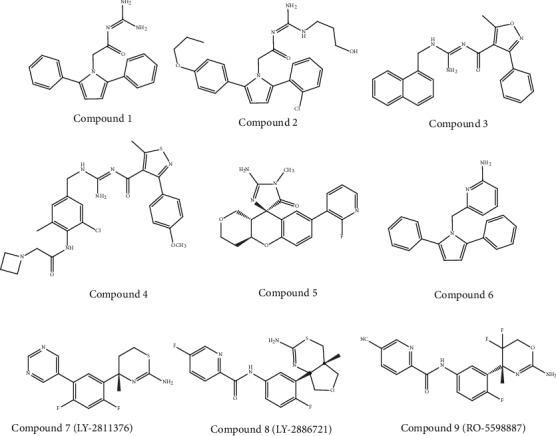
Chemical structure of auspicious molecules (acyl guanidine-, 2-aminopyridine-, aminothiazine-, and aminooxazoline-based inhibitors) targeting *β*-secretase activity.

Interestingly, via using high-throughput screening (HTS), Wyeth (an American pharmaceutical company) has discovered a number of acyl guanidine-based BACE-1 inhibitors [106]. In addition, an X-ray crystal structure of compound 1 (Figure 5) complexed with the catalytic domain of BACE-1 showed that the acyl guanidine moiety forms 4 key hydrogen interactions with the catalytic aspartic acids including Asp228 and Asp32 [106]. Moreover, a structural alteration in BACE-1 upon binding with this compound was detected [106]. In order to improve potency, later substitutions were made to compound 1. On the other hand, compound 2 (Figure 5), comprising a *para*-propyloxyphenyl moiety in the unsubstituted aryl ring and propyl alcohol in the third guanidine nitrogen, permitted an enhancement of about 30-fold in potency as compared to compound 1. However, it has been observed that the 2-chloro group of compound 1 does not play a significant role in the case of potency [106].

Bristol-Myers Squibb (an American pharmaceutical company) has also worked in an acyl guanidine series. They started with hit compound 3 (Figure 5), and developed compound 4 with a good potency against BACE-1 (IC_50_ = 5.0 nM) while remaining inactive against the other aspartyl proteases studied, i.e., pepsin (IC_50_ > 100.000 nM), cathepsin E (CatE), and cathepsin D (CatD) [107]. In rats, the optimized compound 4 was assessed to evaluate its action on the levels of A*β*40 in CSF, the brain, and plasma. A dose-dependent and significant decrease in the level of A*β*40 (about 80%) in plasma was observed, but no substantial decrease in CSF and the brain was attained (<20%). This deficiency of efficacy in CSF and the brain was attributed to the efflux of P-glycoprotein (P-gp). Therefore, further enhancement was essential in order to optimize its PK properties [107]. Interestingly, Array BioPharma (an American biopharmaceutical company) in cooperation with Genentech (a biotechnology corporation-subsidiary of Roche) developed a number of chromane-based spirocyclic acyl guanidine-derived BACE-1 inhibitors leading to compound 5 (Figure 5). Indeed, this compound exhibited a good selectivity towards BACE-1. Additionally, this compound also had the ability to decrease the levels of A*β*40 in CSF from 53% to 63% in rats and cynomolgus monkeys, respectively. Furthermore, this compound exhibited a high efflux ratio of P-gp [108].

### 5.2. 2-Aminopyridine-Based Inhibitors

The discovery of 2-amino heterocycles is regarded as the major progress in the development of small BACE-1 inhibitor molecules. It has been found that these inhibitors commonly possess a better physicochemical profile and can improve *in vivo* efficacy and brain penetration [109]. On the other hand, as an extension of their study on acyl guanidine inhibitors, Wyeth also developed a number of pyrrolyl 2-aminopyridines. It has been suggested that the inhibitors of acyl guanidine are polar due to the presence of the acyl guanidine moiety, which leads to poor permeability (<5%) of BBB. Interestingly, it has been observed that the bioisosteric replacement of the acyl guanidine moiety by an aminopyridine (compound 6) in compound 1 leads to the enhancement of its permeability while preserving the hydrogen-bonding interactions with the two aspartic acids in the catalytic site of BACE-1 [110].

Through X-ray studies, the similarity of the hydrogen interaction in between these 2 compounds was verified, which further established the interaction of the 2-aminopyridine moiety with the aspartic acids Asp32 and Asp228 of the catalytic site of BACE-1 [110]. Interestingly, permeability was shown to be enhanced through the modulation of the total polar surface area. Compound 6 (Figure 5) indeed exhibited a good central drug exposure with a brain-to-plasma ratio of 1.7 in comparison with the 0.04 ratio attained with the acyl guanidine-based compound 1 [110].

### 5.3. Aminothiazine- and Aminooxazoline-Based Inhibitors

A number of aminooxazoline/aminothiazine-based inhibitors have been developed by researchers of different pharmaceutical companies. Unfortunately, out of these compounds, only few of the compounds were able to reach the clinical evaluation stage. Indeed, the first clinical BACE-1 inhibitor which was developed by Eli Lilly (an American pharmaceutical company) is an aminothiazine-based compound, LY-2811376 (compound 7, Figure 5). This pharmaceutical company initiated phase I clinical trials of this compound in 2009. At single doses from 5 to 500 mg as oral capsules, this BACE-1 inhibitor was administered to 61 healthy participants in order to assess its tolerability and safety [111]. It was reported that LY-2811376 exhibited well tolerability with no serious adverse effects [112]. In addition to this, peak concentration was achieved within two hours postdose in the plasma and five hours postdose in CSF. Interestingly, a dose-dependent decrease in the level of A*β*42 and A*β*40 was noticed. Following a single dose of 90 mg, an A*β*40 decrease of 80% was reported within 7 hours and 54% within 12–14 hours, in the plasma and CSF, respectively. On the other hand, in parallel with the phase I clinical trials, a toxicological study was performed in rats. A retinal pathology was observed in these animals at doses ≥ 30 mg/kg, and the pathology was characterized by cytoplasmic buildups of finely granular autofluorescent material dispersed within the retinal epithelium. Therefore, the ongoing clinical studies of LY-2811376 (compound 7) were terminated. Thus, LY-2811376 did not enter into the phase II clinical trial. The aforesaid toxic effect might take place due to the off-target actions of LY-2811376 against other aspartic acid proteases, viz., CatD, as confirmed through a subsequent study by means of LY-2811376 in *BACE-1* knockout mice [112]. After the end of the trial, all the participants were subjected to follow-up study as a safety measure. Luckily, no participants exhibited any clinically significant observations [112].

The second clinically studied compound was an aminothiazine-based inhibitor called LY-2886721 (compound 8, Figure 5). This compound was the first BACE-1 inhibitor that reached phase II trials. In fact, LY-2886721 was studied in six phase I trials to evaluate its pharmacology, safety, and tolerability effects on 150 healthy participants. Interestingly, the administration of this compound for 14 days at a dose of 70 mg/day caused a reduction of CSF A*β*42 by 71% and CSF A*β*40 by 74%. Up to six weeks, no apparent safety concerns were observed with the used dosage [113]. Due to the satisfactory findings obtained in phase I clinical studies, Eli Lilly started a phase II clinical trial in March 2012 to evaluate the tolerability, pharmacodynamic (PD) properties, and safety of LY-2886721 in mild AD patients [114]. In the course of the study, routine safety monitoring identified aberrant elevations in the liver enzyme level in 4 out of 70 patients. Therefore, Eli Lilly terminated the phase II trial and clinical development of LY-2886721. It has been observed that the toxicity exerted by this BACE-1 inhibitor was found to be an off-target action of this compound which was unrelated to the inhibition of BACE-1 [113, 115].

The aminooxazoline-based compound, RG-7129 (RO-5598887, compound 9, Figure 5) was developed by Roche (a Swiss multinational healthcare company). This compound entered into a phase I trial in September 2011. Preclinical studies revealed that it presents high potency against BACE-1 (IC_50_ = 30 nM). Except against BACE-2 (IC_50_ = 40 nM), this compound exhibited selectivity against pepsin, CatE, CatD, and renin (>1000-fold). Between 2012 and 2013, three phase I trials have been completed. Unfortunately, no results were reported in this period. In addition, Roche terminated the development of RG-7129 without providing any clarification [116]. However, in a study published in 2014, Roche started the use of a combination treatment of the anti-A*β* antibody gantenerumab and the BACE-1 inhibitor RG-7129 (compound 9) in transgenic mice, recommending a future clinical assessment of the combination of these two compounds [117].

### 5.4. Aminoimidazole-Based Inhibitors

**Figure 6:**
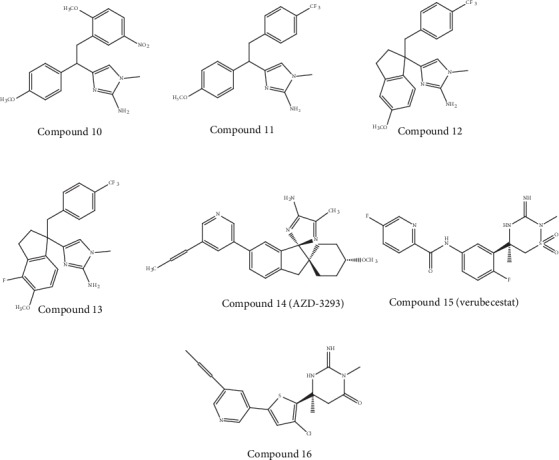
Chemical structure of auspicious molecules (aminoimidazole- and iminothiadiazinane dioxide-based inhibitors) targeting *β*-secretase activity.

It has been revealed through molecular modeling studies that the amino group of the imidazole heterocycle was found to be accountable for the binding with the catalytic aspartic acid residues of BACE-1. Potent inhibitors including compound 10 (Figure 6) were obtained as a result of 2-methoxy-5-nitro substituents on the benzyl subunit. Subsequently, compound 11 (Figure 6) was synthesized, and this latter was found to cause a significant decrease in the P-gp efflux ratio (ER). Therefore, Merck (an American multinational pharmaceutical company) carried out further studies in order to improve this inhibitor's potency. It has been observed that the addition of a conformational constraint in compound 12 (Figure 6) allowed a five-time potency increment because of additional hydrophobic interactions with the flap area of BACE-1 [118]. Furthermore, the addition of a fluorine group in compound 13 (Figure 6) permitted additional hydrophobic interactions as well as a six-times potency increase to an IC_50_ (BACE − 1) value of 63 nM. Moreover, this compound was suggested to exhibit brain penetration capacity due to its decreased P-gp ER of 3.6 [118].

AstraZeneca developed compound AZD-3293 (LY-3314814, Lanabecestat, compound 14 (Figure 6)), which is an aminoimidazole-based compound, and this compound reached phase I trials in 2012 [119]. Currently, the findings obtained from the first two phase I clinical trials are available [120]. These clinical trials involved single ascending dose studies in order to evaluate the doses of 1–750 mg with a food-effect component (*n* = 72). The trials also involved a two-week several ascending dose studies in order to evaluate the doses of 50 or 15 mg once per day or 70 mg once per week in elderly participants (i.e. part 1; *n* = 31) and doses of 150, 50, or 15 mg once per day in individuals with mild-to-moderate AD (i.e. part 2; *n =* 16). Findings obtained from these studies revealed that AZD-3293 was well tolerated up to the highest doses provided. In addition, ≥70% reduction in mean plasma concentrations of A*β*42 and A*β*40 was observed with single doses of ≥5 mg. On the other hand, at the highest dose level studied, prolonged inhibition for up to three weeks has been observed. With the use of multiple doses, a vigorous reduction was observed in A*β* levels of CSF (i.e. ≥76% at ≥50 mg and ≥51% at 15 mg) and plasma (i.e. ≥78% at ≥50 mg and ≥64% at 15 mg). In addition to this, prolonged inhibition was observed even with a once-a-week dosing regimen [120].

Four additional phase I clinical studies of compound 14 were conducted with a total of 175 healthy participants in 2015 and 2016. In these studies, a new tablet formulation was used evaluating possible interaction of this compound with a number of commonly prescribed drugs (including donepezil, simvastatin, midazolam, dabigatran, and warfarin) in the elderly [121]. Presently, Eli Lilly and AstraZeneca are sponsoring the two phase III clinical trials of AZD-3293 (compound 14) [119]. At daily doses of 20 or 50 mg for 18 to 24 months, these placebo-controlled, double-blind, multicenter, randomized studies are evaluating the disease-modifying potential of AZD-3293 in over 4,000 individuals with early and mild AD [119, 120].

### 5.5. Iminothiadiazinane Dioxide-Based Inhibitors

A series of iminothiadiazinane dioxide-based inhibitors represented by verubecestat (compound 15, Figure 6) has been developed by Merck. Interestingly, to explore a new intellectual property space and improve binding affinity, the starting point was an iminopyrimidinone scaffold (compound 16, Figure 6) which was modified [105]. The compound 15 (verubecestat) exhibited good PD/PK properties in animal models. Furthermore, this compound caused sustained and marked decrease in the levels of CSF A*β*40 in cynomolgus monkeys (i.e., 81% and 72% decrease at 10 and 3 mg/kg, respectively). In telemetered monkeys, this compound did not exhibit any action on the QT interval in a single-dose cardiovascular study. On the other hand, it caused no stimulation of expression of CYP1A2 or CYP3A4 in human hepatocytes [122].

In 2011, compound 15 entered into phase I trials, where its PD, PK, and safety were assessed in healthy participants as well as in individuals with mild-to-moderate AD. In the case of AD and healthy individuals, both the multiple and single doses were well tolerated and decreased A*β* levels in the CSF. Furthermore, in any of the phase I trials, no alterations were observed in hair or skin pigmentation due to the inhibition of BACE-2. Pigmentation alterations became apparent for prolonged treatment times [122]. The compound 15 advanced to phase II/III trials in 2012, and the trials involved individuals with mild-to-moderate AD (i.e. the EPOCH trial). Additionally, a phase III trial of this compound started in 2013 for prodromal AD (i.e. the APECS trial) [109].

Because of the lack of efficacy as well as the presence of marked neuronal and synaptic losses, the EPOCH trial was terminated in February 2017 [123]. On the other hand, the APECS trial was also terminated in February 2018. Furthermore, Merck excluded verubecestat from its research pipeline. As compared to the placebo group, volunteers who were administered verubecestat scored worse on the cognitive test Alzheimer's Disease Cooperative Study-Activities of Daily Living (ADCS-ADL). More studies are essential to understand the reason of this negative effect [124, 125].

## 6. Anti-Alzheimer's Molecules Targeting *γ*-Secretase

It has been found that unlike BACE1, GS has demonstrated to be a highly manageable target for AD, at least in terms of the development of orally bioavailable GS inhibitors (GSIs) with brain-penetrating capacity [66]. For mouse and human brains, GSIs have been found to reduce the production of A*β*. In the case of APP mouse models, the deposition of A*β* was found to be decreased due to the chronic administration of GSIs [126–129]. So far, several orally bioavailable GSIs presenting brain-penetrant properties have been found. Nevertheless, a vital problem in the development of GSIs is target-based toxicity. This target-based toxicity was observed on the basis of preclinical studies [71]. Along with APP, a number of other type 1 transmembrane proteins are also processed by the GS enzyme. In this regard, for instance, GS processing of Notch1 is essential for Notch signaling [130]. In mice, *PESN1* deletion is embryonically lethal. Furthermore, these mice exhibit a phenotype—protuberant brain and skeletal abnormalities—that is also obvious in mice lacking the notch [131, 132]. In addition to this, treatment with nonselective GSIs can lead to various Notch inhibition-related toxicities, including (but not limited to) abnormalities of the integumentary, immune, and gastrointestinal systems [133–135].

In phase III trials, semagacestat (a GS inhibitor) failed to attain the primary goals due to observed worsening symptoms in some patients [85]. The study of avagacestat was terminated due to the serious adverse events observed in a phase II trial including nonmelanoma skin cancer, dose-dependent glycosuria, and cerebral microbleeds. Interestingly, EVP-0962, the selective, small molecule, orally available GS modulator (GSM), decreases the production of A*β*_1-42_ via shifting the cleavage of APP toward the generation of less toxic and shorter A*β*, without affecting the cleavage of Notch [136]. On the other hand, the naturally occurring cyclic sugar alcohol NIC5-15 has the ability to modulate the activity of GS to lower the production of A*β*. NIC5-15 is currently in phase II clinical trials of Alzheimer's treatments [137].

### 6.1. NSAID-Derived *γ*-Secretase Modulators

The first generation of GSMs was discovered from an epidemiological study. This study reported a decreased prevalence of AD among the individuals who were administered nonsteroidal anti-inflammatory drugs (NSAIDs) [138]. Subsequently, Weggen et al. [138] stated that NSAIDs reduced the level of A*β*42 along with the increment of an A*β*38 isoform, which further indicated that the activity of GS can be modulated by NSAIDs without causing a marked disturbance of Notch cleavage or other APP-processing mechanisms [138]. The aforesaid alteration in the pattern of cleavage could be explained by several processes including an elevation in the GS's cleavage activity (defined by the catalytic constant, *κ*_cat_) or a reduction in the possibility of releasing longer A*β* from the enzyme-substrate complex (defined as a dissociation constant, *κ*_d_). Chávez-Gutiérrez et al. [139] through the use of CTF 99 as a substrate showed that the ratios of A*β*38/A*β*42 and A*β*40/A*β*43 were reduced via all the *PESN* mutations studied, further recommending a weakening of the 4 cleavage cycles of GS (GS epsilon-cleavage). In addition to this, Okochi et al. [140], using A*β*42 as a substrate, estimated the kinetic constants for the *γ* cleavage. These researchers also stated that GSMs elevated *κ*_cat_ and reduced *κ*_d_, whereas mutations of PS lead to the opposite effect. Collectively, these findings indicated that the GS modulation effect might be a useful technique to reverse the action of PS mutations through the restoration of the normal ratios of the formed A*β*, in this manner modifying the progression and pathology of the disease [141].

**Figure 7:**
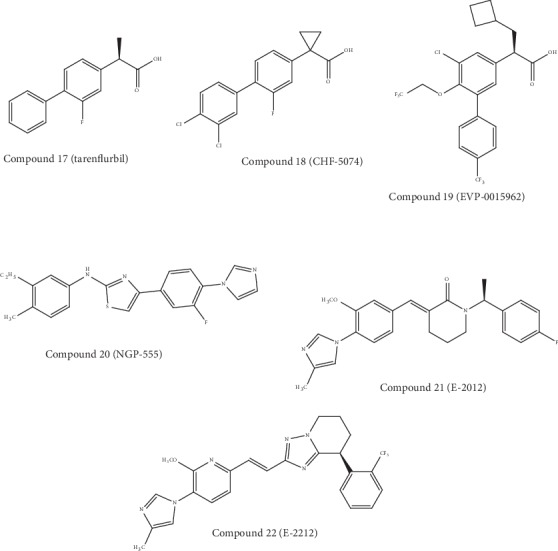
Chemical structure of auspicious molecules targeting *γ*-secretase activity.

However, the use of NSAID as GSMs can induce some risks of renal and gastrointestinal toxicity because of the effects of NSAIDs against the cyclooxygenase 1 (COX-1) enzyme. This aforesaid problem of NSAID is compromising its usage for long-term therapeutic solutions [142]. Luckily, the activity of COX-1 inhibition was found to be not dependent on the activity of GS modulation. For example, flurbiprofen is a COX-1 inhibitor which was administered as a racemate for clinical trials. Nevertheless, tarenflurbil (compound 17, Figure 7) exhibited a decreased activity of COX-1 inhibition while preserving its action as a GSM [142]. This latter compound has entered into phase III trials. However, data exhibited no differences between the placebo and tarenflurbil. The cause of this failure was attributed to its inadequate PD properties. In fact, weak CNS penetration of tarenflurbil was formerly stated in preclinical studies in rodents, with a CSF/plasma ratio of 1.3%. The clinical development of tarenflurbil was terminated in view of these unsatisfactory findings [143].

Subsequently, 2 NSAID carboxylic acid derivatives were developed by Chiesi Farmaceutici (an Italian family-controlled global pharmaceutical company) (CHF-5074, compound 18, Figure 6) and FORUM Pharmaceuticals (EVP-0015962, compound 19, Figure 7) and also studied in clinical studies. Based on the scaffold of tarenflurbil (compound 17), Chiesi Farmaceutici developed CHF-5074 (compound 18). Interestingly, there was complete cessation of COX inhibition (at 100 *μ*M and 300 *μ*M) by a cyclopropyl group. It has been observed that the inhibition of A*β*42 generation displayed a 7-fold improvement due to the introduction of chlorine substituents on the terminal phenyl ring as compared to tarenflurbil (compound 17) [144]. It was proposed that the carboxylic acid group interacts with a lysine residue of APP situated close to the membrane interface, with the lipophilic substituents playing a role as membrane anchors [145]. In 2012, EVP-0015962 (compound 19) entered into phase II trials, but findings were not posted publicly on ClinicalTrials.gov.

### 6.2. Non-NSAID-Derived *γ*-Secretase Modulators

In 2004, NeuroGenetic Pharmaceuticals (an American pharmaceutical company) developed one of the first GSM classes not presenting a carboxylic acid moiety (non-NSAID), which further lead to the discovery of NGP-555 [146]. NGP-555 (compound 20, Figure 7) exhibited a reduction in CSF A*β*_42_ levels between 20 to 40% and an elevation of the shorter forms in rodent studies. In addition to that, this compound confirmed neuroprotection from cognitive impairment in two independent mice experiments by employing various learning and memory tasks [147]. In 2015, NGP-555 was taken into phase I trials. In January 2017, NeuroGenetic Pharmaceuticals revealed that this compound exhibited well-tolerability and safety in healthy participants [148]. However, detailed findings and future clinical trials with this compound have not been revealed yet. Interestingly, the work on the cinnamide series from Eisai Pharmaceuticals leads to the discovery of the clinical compounds E-2012 (compound 21, Figure 7) and E-2212 (compound 22, Figure 7) [146]. In a dose-dependent manner, E-2012 reduced the levels of A*β*42 and A*β*40 in rat plasma, brain, and CSF *in vivo*. It has been reported that the IC_50_ values of E-2012 for A*β*42 and A*β*40 were 92 and 330 nM, respectively [149]. On the other hand, E-2012 markedly reduced the levels of A*β*42 in rat CSF by 47.2% and 16.6% at doses of 30 and 10 mg/kg, respectively. Furthermore, E-2012 caused a reduction of the levels of A*β*42 and A*β*40 and elevated the levels of shorter A*β*s (i.e. A*β*37 and A*β*38), without altering the total amount of A*β* [150]. E-2012 (compound 21) was moved into the clinical trials in 2006 as the first noncarboxylic acid compound. Furthermore, during a phase I clinical trial, this compound exhibited efficacy in terms of decreasing the A*β*42 levels in plasma (~50%) [151]. Nevertheless, in parallel to the phase I clinical trial, lenticular opacity was seen in a high-dose group of a 13-week preclinical safety study in rats leading to the termination of the clinical trial. However, ocular toxicity was not observed in monkeys, when follow-up studies were done with up to the highest tolerated dose for E-2012 [152]. Nonetheless, Eisai Pharmaceuticals decided to further develop the improved compound E-2212 (compound 22) [141].

In 2010, E-2212 (compound 22) was taken into a phase I clinical trial. This compound exhibited a similar pharmacological profile as E-2012 (compound 21) as well as improved safety profile, along with no clinically important ophthalmologic findings [153]. In addition to this, the PD response measured in the plasma elevated with the dose and was revealed to perform a 54% decrease in the level of A*β*42 at the dose of 250 mg. To date, Eisai Pharmaceuticals has not revealed any reports regarding the further development of E-2212 (compound 22). Furthermore, there is no update regarding possible new studies on ClinicalTrials.gov. The anticipated structure of E-2212 (compound 22) containing a high cLogP (5.5), high molecular weight (480 g/mol), and four aromatic rings [141] might have hardened its further development because of its poor drug-like properties.

## 7. Anti-Alzheimer's Molecules Targeting A*β* Accumulation

**Figure 8:**
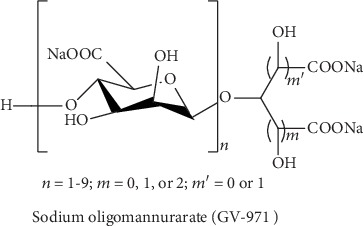
Chemical structure of sodium oligomannurarate targeting A*β* accumulation.

Multiple enzymes can cause degradation of A*β* and have been considered for new drug development [154, 155]. The newly developed anti-AD drug sodium oligomannurarate (GV-971, Figure 8) by Shanghai Green Valley Pharmaceuticals can bind with several sites of amyloid and can cause destabilization and inhibition of A*β* aggregation, which can eventually elevate the clearance of A*β* [156]. The activity of GV-971 was also evaluated in individuals with mild-to-moderate AD in phase III clinical trials [157]. An alteration in the Alzheimer's Disease Assessment Scale-Cognitive Subscale (ADAS-Cog) score was observed as the main endpoint of that clinical study. Additionally, other findings of this study also indicate the significant beneficial effects of GV-971 on cognitive functions. The China National Medical Product Administration (NMPA) provisionally permitted (on November 2, 2019) the use of GV-971 to treat individuals with mild-to-moderate AD [157].

The use of immunotherapy to clear A*β* also seems a reasonable approach [158]. Multiple clinical studies are carried out with monoclonal antibodies that target A*β*. Aducanumab is a monoclonal antibody that targets A*β* aggregations [159]. Nonetheless, Biogen and Eisai declared on March 21, 2019, that they would stop the 221AD301 phase III study of aducanumab (BIIB037) in individuals with an early stage of AD (ENGAGE) and the 221AD302 phase III study of aducanumab (BIIB037) in individuals with an early stage of AD (EMERGE). This decision was taken according to the results of an interim analysis anticipating that ENGAGE and EMERGE were likely to miss their main endpoints [160]. Furthermore, Biogen reported on April 24, 2019, that it would not start the projected phase III secondary prevention program with aducanumab and terminated further studies. However, Biogen declared on October 22, 2019, that the interim analysis was incorrect. Moreover, following analysis of a larger data set, Biogen rather revealed that EMERGE had met its main endpoint. Later, Biogen declared the plan to apply for regulatory approval in early 2020 for aducanumab in the US [160].

Crenezumab is an anti-A*β* monoclonal antibody with a specific affinity towards all fibrillary, oligomeric, and pentameric amyloids [161]. Crenezumab is being assessed in a clinical trial of crenezumab versus placebo to assess the safety and efficacy in individuals with prodromal-to-mild AD (CREAD). Nevertheless, Roche terminated both the CREAD 1 and CREAD 2 studies. Following an interim analysis, it was indeed observed that the trial was less likely to achieve its primary endpoint of reducing the decrease on the Clinical Dementia Rating Sum of Boxes (CDR-SB) data [162].

## 8. Anti-Alzheimer's Molecules Targeting the Phosphorylation Signaling in APP Processing

Like A*β*, hyperphosphorylated tau has been detected in the brains of individuals with AD; therefore, protein kinase inhibitors can play an important role to reduce AD by primarily targeting pathogenic tau [163, 164]. Hyperphosphorylation can cause loss of tau solubility and can lead to paired helical filament (PHF) formation, which can further cause NFT formation [165–168]. The glycogen synthase kinase (GSK)3*β* has a significant contribution to the phosphorylation of tau. Furthermore, GSK3*β* causes phosphorylation of around 31% of the pathological phosphorylation sites of tau [166]. GSK3*β* activity can be increased by toxic A*β*, which can eventually increase the production of A*β* through the phosphorylation of tau [169, 170]. Thus, GSK3*β* forms a relationship in between tau pathology and A*β* toxicity [166]. The cyclin-dependent kinase 5 (CDK5) is another vital tau protein kinase associated with pathophysiological tau phosphorylation. Typically, p35 (i.e., the CDK5 regulating protein) is found truncated to 25 amino acids in the brain of individuals with AD. Interestingly, hyperphosphorylated tau dissociates from the microtubules and produces NFTs on CDK5 stimulation by p25 [171, 172]. The activity of CDK5 can also cause NFT phosphorylation [173, 174].

**Figure 9:**
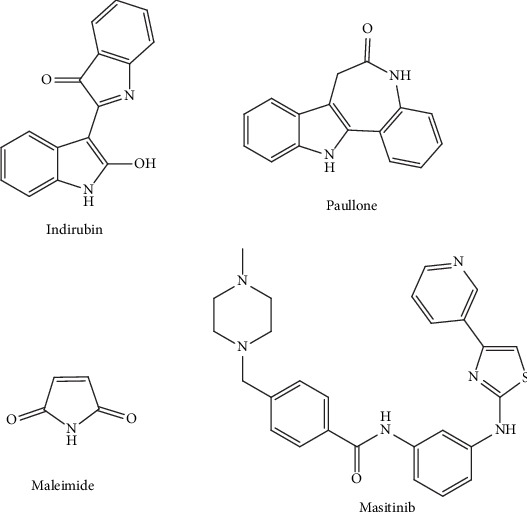
Chemical structure of indirubin, paullone, maleimide, and masitinib targeting the phosphorylation signaling in APP processing.

It has been observed that lithium can inhibit GSK3*β* and lead to a decreased rate of tau phosphorylation, which ultimately averted tau pathology in animal models [175]. However, no improvement in cognitive functions was observed, when used in individuals with early-stage of AD [176]. These findings are associated with unaltered biomarkers of AD in CSF of the individuals including toxic A*β*, phosphorylated tau, and total tau [176]. Multiple GSK3*β* inhibitors are also being developed that belong to the indirubin, paullone, and maleimide (Figure 9) families. Nevertheless, none of these inhibitors have entered into clinical studies until now. The cytotoxic effects of these inhibitors are regarded as the main reasons for their failure [177]. In another study, administration of the CDK5 inhibitor was reported to decrease the levels of A*β* and increase the levels of phospho-tau in very old and nontransgenic mice [178]. However, in the elderly mice, CDK5 inhibitors might not be useful in targeting tau phosphorylation [178].

Fyn physically associates with tau and can cause phosphorylation of tyrosine residues, such as Tyr18, close to the amino terminus [179–182]. Tyr18 is also phosphorylated in NFTs in the brains of AD individuals, which denotes a probable clinical relevance [179]. Masitinib (Figure 9) is an inhibitor of tyrosine kinase. Masitinib shows selectivity towards c-Kit, platelet-derived growth factor receptor, and, to a lesser extent, Lyn and Fyn. Masitinib was studied in a 24-week, placebo-controlled, randomized, phase II study involving 34 mild-to-moderate AD patients. Masitinib was found to be reasonably well tolerated and was linked with improved cognitive functions at 12 and 24 weeks, therefore suggesting inhibition of tyrosine kinase as a therapeutic approach for AD. There is also an ongoing phase III clinical study to assess the safety and efficacy of various doses of masitinib as compared to placebo [183].

## 9. Conclusions

For rational anti-AD drug design, the characterization and identification of the secretase enzymes that cleave APP provides a molecular framework. In terms of physiological importance, the three secretases have extensively been studied. Thus, we now have the appropriate understanding and knowledge regarding the potential adverse events related to the use of drugs that modulate the secretases effects. Currently, multiple compounds targeting each of the 3 secretases are under clinical studies. The use of high throughput and virtual screening, followed by chemical optimization is trying to accommodate some physicochemical nuances and discover new compounds that will have the required safety and efficacy to modify the progression of AD. Henceforth, based on the currently available information, *α*-, *β*-, and *γ*-secretases can be considered as safe and efficacious targets for the reduction of AD pathology. Collectively, the future of AD will depend on the development of safe, potent, and selective compounds that will have the ability to delay the development and progression of AD. Furthermore, it is essential to identify the specific biomarkers which will allow an effective and early pharmacological intervention for AD.
